# A comparison of patients with neck abscesses caused by esophageal foreign body impaction vs. inflammatory disease: a retrospective study

**DOI:** 10.1186/s12893-022-01860-0

**Published:** 2022-12-02

**Authors:** Xuewei Wang, Feng Xu, Danzheng Liu, Xuemei Luo, Xu Zhou, Xinsheng Huang, Na Shen

**Affiliations:** Department of Otolaryngology-Head & Neck Surgery, Zhongshan Hospital, Fudan University, 180 Fenglin Road, Shanghai, 200032 People’s Republic of China

**Keywords:** Neck abscess, Foreign body impaction, Inflammation

## Abstract

**Objectives:**

During clinical practice, we have detected a few cases of neck abscesses in patients diagnosed with esophageal foreign body impaction (EFB) but without the primary inflammatory disease. However, we do not know if neck abscesses caused by an inflammatory source are more like to be associated with a more severe progression or poorer prognosis. In this study, we aimed to identify differences between these two groups of patients by comparing progression and prognosis.

**Materials and methods:**

We retrospectively reviewed all patients who underwent neck abscess incisions between January 2011 and March 2022 and divided these patients into two groups: an EFB group and an inflammation group. Data were described by percentages, means, and standard deviations (SDs). Fisher's precision probability test was used to compare differences between the EFB and inflammation groups. Categorical variables were analyzed by Pearson's Chi-squared test. In addition, three factors including hospital days, intensive care unit (ICU) stay, and drainage-tube removal time were used for multivariate analysis to identify independent correlations separately.

**Results:**

We enrolled a total of 33 patients with neck abscesses who received surgical incisions; the EFB group included 14 (42%) cases, while the inflammatory group included 19 (58%) cases. No significant differences were identified between the two groups in terms of surgery type (with or without mediastinotomy) and postoperative management (negative pressure drainage or postoperative irrigation). There were no significant differences between the two groups in terms of hospital stay, the timing of drainage-tube removal, the risk of ICU admission, and the probability of receiving intubation and tracheotomy. The incidence rate of esophageal perforation differed significantly between the two groups (p < 0.001). However, there were no significant differences in terms of other preoperative or postoperative comorbidities. The multivariate analysis revealed that the application of mediastinotomy (HR = 0.216 [0.049, 0.963]; p = 0.044) was correlated with a longer stay in the hospital. The time from symptoms to surgery was associated with a longer drainage tube removal time (HR = 0.392 [0.159, 0.967]; P = 0.042) and longer ICU stay (OR = 79.754[1.513, 4203.182]; P = 0.03).

**Conclusion:**

Patients with neck abscesses associated with EFB and inflammation received the same therapeutic management, and there were no significant differences between these two groups in terms of prognosis. Furthermore, esophageal perforation was found to be irrelevant to the aggravation of neck abscesses, and there was no need for additional surgery to repair a perforated esophagus in patients with neck abscesses.

*Level of evidence:* Retrospective cohort (2b).

## Introduction

Neck abscesses represent the final stage of deep neck infections that manifest as cellulitis during the early stages of the disease. On account of the special anatomy of the head and neck, infections can spread through anatomical spaces and penetrate the fascial planes, thus causing extensive abscesses in deeper regions; these infections can result in fatal complications such as airway obstruction and sepsis [1]. It is now commonly acknowledged that a patient with a deep neck infection within the cellulitis stage can be completely cured by single therapy intravenous antibiotics. However, an immediate surgical procedure is inevitable once the abscess is formed; otherwise, the patient may suffer from irreversible consequences [2]. Therefore, deep neck infections should be taken seriously.

In most published research, the causative factors for deep neck infections were focused on inflammatory diseases, mainly throat and odontogenic infections, as well as peritonsillitis, salivary gland infections, and secondary infections caused by neoplasm and penetrating or blunt trauma. Factors of odontogenic origin were identified in 30% to 50% of deep neck infections [3–5]. However, in our clinical practice, we have identified several neck abscesses in patients with esophageal foreign body impaction (EFB) but without the primary inflammatory disease. These findings indicated that esophageal foreign body impaction might represent a key factor for the formation of neck abscesses; however, this hypothesis has yet to be addressed.

EFB is a disease that is commonly identified in the otolaryngology department; these patients often require emergency treatment, especially following the accidental swallowing of foreign objects [6]. According to statistics, 24,529 cases of upper gastrointestinal foreign body impaction were reported in China between 2010 and 2015; most of these foreign bodies were identified in the esophagus, especially the esophageal entrance [7]. Without timely and appropriate treatment, EFB may cause esophageal perforation, thus speeding the neck infection and, ultimately, the formation of neck abscesses.

Due to the lack of relevant studies, the majority of patients with EFB-related neck abscesses receive the same therapeutic management as those with inflammation-related neck abscesses. However, it is not known whether neck abscesses caused by an inflammatory source are more like to be associated with a more severe progression or poorer prognosis or whether EFB-sourced neck abscesses require two-stage surgeries. In this study, we aimed to investigate differences between these two groups of patients by comparing progression and prognosis.

## Material and methods

We retrospectively reviewed all patients with neck abscesses who underwent surgical incision drainage between January 2011 and March 2022. This research study was approved by the Ethics Committee of Zhongshan Hospital Affiliated to Fudan University (B2022-400R). Written informed consent was obtained from all patients included in this study. The study protocol conformed to the ethical guidelines of the 1975 Declaration of Helsinki as reflected in a priori approval by the Institution's human research committee.

**Fig. 1:**
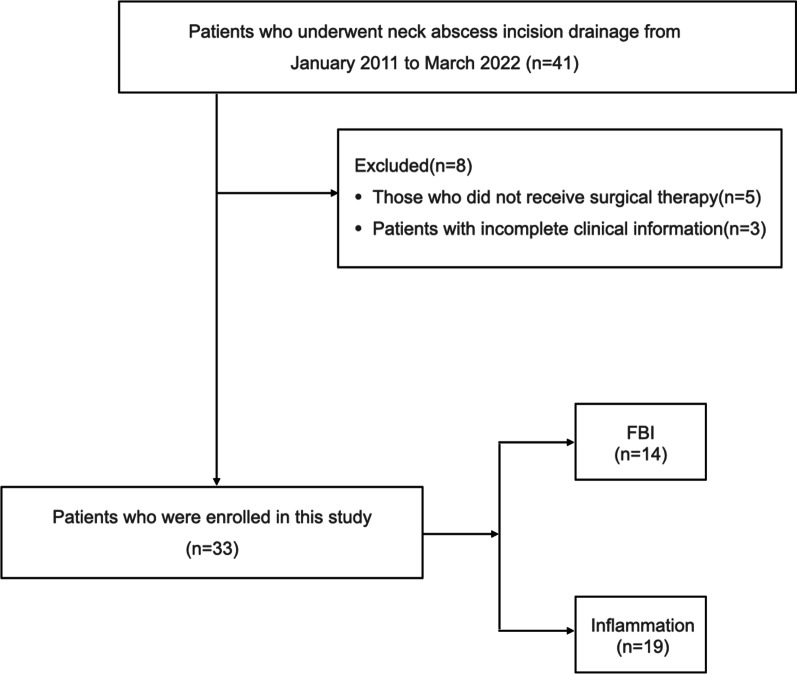
Flowchart of the study patients

In this study, we enrolled all cases with a diagnosis of neck abscess by clinical assessments and contrast-enhanced computed tomography (CT); each case was confirmed by subsequent surgery. Patients who did not receive surgical therapy or had an incomplete dataset of clinical information were excluded (Fig. 1). Then, the enrolled patients were divided into two groups according to the causative factors involved: esophageal foreign body impaction and inflammation. Next, we investigated the correlation between these grouping factors and the occurrence of neck abscesses. We also ensured that there were no other inflammatory inducements in the EFB cohort and no history of any form of EFB in the inflammation cohort. By searching medical records databases, we collated a range of information related to each patient, involving age, sex, hospital stay, the duration of symptoms prior to visiting and surgery, mediastinotomy, ultrasound puncture, negative pressure drainage, post-operative irrigation, the time to tube removal, intubation and tracheotomy, bacterial culture, pre-operative comorbidities, type of foreign body, post-operative comorbidities, intensive care unit (ICU) stay and length of stay (LOS).

## Statistical analyses

Data were illustrated as percentages, means, and standard deviations (SDs). Fisher's precision probability test was used to compare differences between the EFB and inflammation groups. Categorical variables were analyzed by Pearson's Chi-squared test. We also selected three factors (hospital days, ICU stay, and the time of drainage tube removal) for multivariate analysis to identify their independent correlations separately. In the regression models, all potential covariates were input into the models. Furthermore, two survival analyses were conducted to evaluate differences between the two groups in terms of hospital days and drainage time. Due to the limitations imposed by this retrospective study, the sample size varied in different statistical analyses depending on the availability of data. Data were analyzed using STATA 17.0 software (StataCorp, TX, USA), and p < 0.05 was considered statistically significant.

## Results

### Patient demographics

In this study, we included 33 patients (21 males and 12 females) with neck abscesses who received surgical incisions (Table 1); the mean age was 58.4 ± 2.5 years (range: 25 to 80 years). The mean duration of symptoms before surgery was 10.1 ± 0.9 days, and the mean hospitalization time was 21.8 ± 2.1 days. Five patients received preoperative ultrasound-guided puncture and drainage. Mediastinotomy was performed on 11 cases (33.3%) during surgery. The majority of patients (84.9%) received negative pressure drainage, while five patients received post-operative irrigation simultaneously. One patient received post-operative irrigation without negative pressure drainage. The mean time of drainage tube removal was 11.9 ± 1.4 days. In total, 27.3% and 18.2% of patients experienced intubation and tracheotomy, respectively. Of note, a total of 21 patients (63.6%) were sent to ICU after surgery.

**Table 1:** Descriptive information by different cause

Total (n = 33)	Esophageal Foreign Body (n = 14)	Inflammatory (n = 19) p-value	
Gender (%)			0.506
Male 21 (63.6)	8 (57.1)	13 (68.4)	
Female 12 (36.4)	6 (42.9)	6 (31.6)	
Age (Year), Mean ± SD 58.4 (2.5)	53.9 (4.1)	61.7 (3.0)	0.126
Hospital stay (days), Mean ± SD 21.8 (2.1)	19.5 (3.1)	23.5 (2.9)	0.359
Symptom-surg, Mean ± SD 10.1 (0.9)	9.7 (1.4)	10.4 (1.1)	0.698
Mediastinotomy (%)			0.618
Yes 11 (33.3)	4 (28.6)	7 (36.8)	
No 22 (66.7)	10 (71.4)	12 (63.2)	
Ultrasound puncture (%)			0.075
Yes 5 (15.6)	4 (28.6)	3 (5.6)	
No 27 (84.4)	10 (71.4)	17 (94.4)	
Negative pressure drainage (%)			0.388
Yes 28 (84.9)	11 (78.6)	17 (89.5)	
No 5 (15.1)	3 (21.4)	2 (10.5)	
Post operative irrigation (%)			0.158
Yes 6 (18.2)	1 (7.1)	5 (26.3)	
No 27 (81.8)	13 (92.9)	14 (73.7)	
Tube remove (days), Mean ± SD 11.9 (1.4)	10.4 (1.7)	13.1 (2.0)	0.350
ICU (yes,%) 21 (63.6)	7 (50.0)	14 (73.7)	0.162
Intubation (%)			0.518
Yes 9 (27.3)	3 (21.4)	6 (31.6)	
No 24 (72.7)	11 (78.6)	13 (68.4)	
Tracheotomy (%)			0.618
Yes 6 (18.2)	2 (14.3)	4 (21.0)	
No 27 (81.8)	12 (85.7)	15 (79.0)	
Pre-operative comorbidities (yes,%)			
Hypertension 9 (27.3)	3 (21.4)	6 (31.6)	0.518
Diabetes mellitus 10 (30.3)	3 (21.4)	7 (36.8)	0.341
Esophageal perforation 16 (48.5)	14 (100)	2 (10.5)	< 0.001
Mediastinal abscess 14 (42.4)	5 (35.7)	9 (47.4)	0.503
Post-operative comorbidities (yes,%)			
Hoarseness 1 (3.0)	0 (0)	1 (5.3)	0.383
Renal dysfunction 1 (3.0)	0 (0)	1 (5.3)	0.383
Heart dysfunction 1 (3.0)	0 (0)	1 (5.3)	0.383
Liver dysfunction 2 (6.1)	1 (7.1)	1 (5.3)	0.823
Dermapostasis 2 (6.1)	0 (0)	2 (10.5)	0.210
Coma 1 (3.0)	0 (0)	1 (5.3)	0.383
Pneumonia 5 (15.1)	2 (14.3)	3 (15.8)	0.905

Esophageal perforation (48.5%), hypertension (27.3%), diabetes (30.3%), and mediastinal abscess (42.4%) were the most common pre-operative comorbidities. With regards to post-operative comorbidities, pneumonia (15.1%) was associated with the highest risk, although dermapostasis (6.1%), liver dysfunction (6.1%), heart dysfunction (3%), renal dysfunction (3%), coma (3%) and hoarseness (3%) were also observed.

### Comparison of the EFB and inflammation cohorts

The EFB group included 14 (42%) cases, while the inflammatory group included 19 (58%) cases. The etiologies of abscesses in the inflammatory group were mainly attributed to throat infection, including acute pharyngitis (15.8%), acute epiglottitis (15.8%), and acute laryngitis (10.5%). Odontogenic infection (15.8%) was the second major etiology, followed by sialadenitis like submaxillaritis (10.5%) and parotitis (5.3%). The foreign body type in the EFB group were mainly animal bones (92.8%), especially fish bones (71.4%; Table 2). There were no significant differences between the two groups in terms of age and sex. The mean length of hospital stay for EFB patients was 19.5 ± 3.1 days; this was less than 23.5 ± 2.9 days for the inflammatory patients. Similarly, the duration of symptoms to surgery time and tube removal time in the inflammatory group were both slightly longer than the EFB group (10.4 ± 1.1 days vs. 9.7 ± 1.4 days; 13.1 ± 2.0 days vs. 10.4 ± 1.7 days, respectively). Moreover, the inflammatory group accounted for twice the proportion of ICU admissions when compared with the EFB group. However, none of these differences were statistically significant (p > 0.05).

**Table 2:** Etiologies for neck abscess

Etiologies of inflammatory group (%)		Etiologies of ^*^EFB group (%)	
Odontogenic infection	3 (15.8)	Fish bone impaction	10 (71.4)
Acute pharyngitis	3 (15.8)	Chicken bone impaction	3 (21.4)
Acute epiglottitis	3 (15.8)	Metal impaction	1 (7.1)
Acute laryngitis	2 (10.5)		
Submaxillaritis	2 (10.5)		
Peritonsillitis	1 (5.3)		
Trauma	1 (5.3)		
Thyroiditis	1 (5.3)		
Parotitis	1 (5.3)		
Superior mediastinal mass	1 (5.3)		
Unknown	1 (5.3)		

^*^The patients of EFB group all underwent endoscopic removal and 3 failed

The incidence rate of esophageal perforation differed significantly between the two groups (p < 0.001). All 14 cases in the EFB group had esophageal perforation; this was compared with only two cases in the inflammatory group. Except for esophageal perforation, other preoperative comorbidities (hypertension, diabetes, and mediastinal abscess) were more likely to occur in the inflammatory group with a two-fold higher risk. Similarly, the risk of postoperative comorbidities was also higher in the inflammatory group, while many complications were detected only in inflammatory cases, such as dermapostasis (p = 0.21), coma (p = 0.38), hoarseness (p = 0.38), renal dysfunction (p = 0.38) and heart dysfunction (p = 0.38). However, these were not statistically significant (p > 0.05). In addition, there were no significant differences with regard to various criteria in the type of surgery (with or without mediastinotomy), postoperative management (negative pressure drainage and postoperative irrigation), or the management of intubation and tracheotomy.

### Multivariate analysis

We generated a multivariate logistic regression analysis model and two multivariate cox regression analysis models to search for factors that were independently associated with ICU stay (Table 3), hospital stay (Table 4), and the time for drainage tube removal (Table 5). A variety of variables obtained from clinical records and demographic information were regarded as covariates. The application of mediastinotomy (HR = 0.216 [0.049, 0.963]; p = 0.044) was correlated with a longer stay in the hospital. The time from symptoms to surgery was associated with a longer drainage tube removal time (HR = 0.392 [0.159, 0.967]; P = 0.042) and longer ICU stay (OR = 79.754[1.513, 4203.182]; P = 0.03). Other covariates were not significant.

**Table 3:** Multi-variable logistic regression for the risk factors of ICU stay

Risk factors	OR	p-value	[95% Conf.	Interval]
Different cause	0.142	0.349	0.002	8.444
Esophageal perforation	0.925	0.972	0.013	68.297
Mediastinal abscess	11.967	0.155	0.391	366.664
Age	6.060	0.357	0.131	280.735
Mediastinotomy	11.760	0.444	0.021	6490.270
Diabetes	1.743	0.796	0.026	116.816
Hypertension	0.048	0.266	0.000	10.041
Time to Surgery	79.754	0.030	1.513	4203.182

**Table 4:** Multi-variable cox regression for the risk factors of hospital days

Risk Factors	HR	p-value	[95% Conf.	Interval]
Different cause	0.654	0.617	0.124	3.458
Esophageal perforation	1.419	0.677	0.274	7.348
Mediastinal abscess	1.973	0.297	0.551	7.069
Time to surgery	1.374	0.571	0.458	4.123
Age	1.947	0.221	0.670	5.661
Cardiac-insufficiency	0.254	0.333	0.016	4.077
Liver-insufficiency	0.238	0.450	0.006	9.898
Renal-insufficiency	1.000			
Dermapostasis	0.355	0.605	0.007	18.025
Coma	1.000			
Pneumonia	0.844	0.766	0.277	2.571
Tracheotomy	0.475	0.295	0.118	1.913
Endotracheal-intubation	0.352	0.088	0.106	1.168
Mediastinotomy	0.216	0.044	0.049	0.963

**Table 5:** Multi-variable cox regression for the risk factors of drainage-tube removing time

Risk Factors	HR	p-value	[95% Conf	Interval]
Different Cause	2.362	0.283	0.492	11.334
Esophageal Perforation	0.549	0.466	0.109	2.751
Mediastinal Abscess	0.740	0.547	0.278	1.970
Time to Surgery	0.392	0.042	0.159	0.967
Age	0.688	0.391	0.292	1.619
Tracheotomy	0.466	0.191	0.148	1.464
Mediastinotomy	0.422	0.114	0.145	1.230

### Survival analysis

**Fig. 2:**
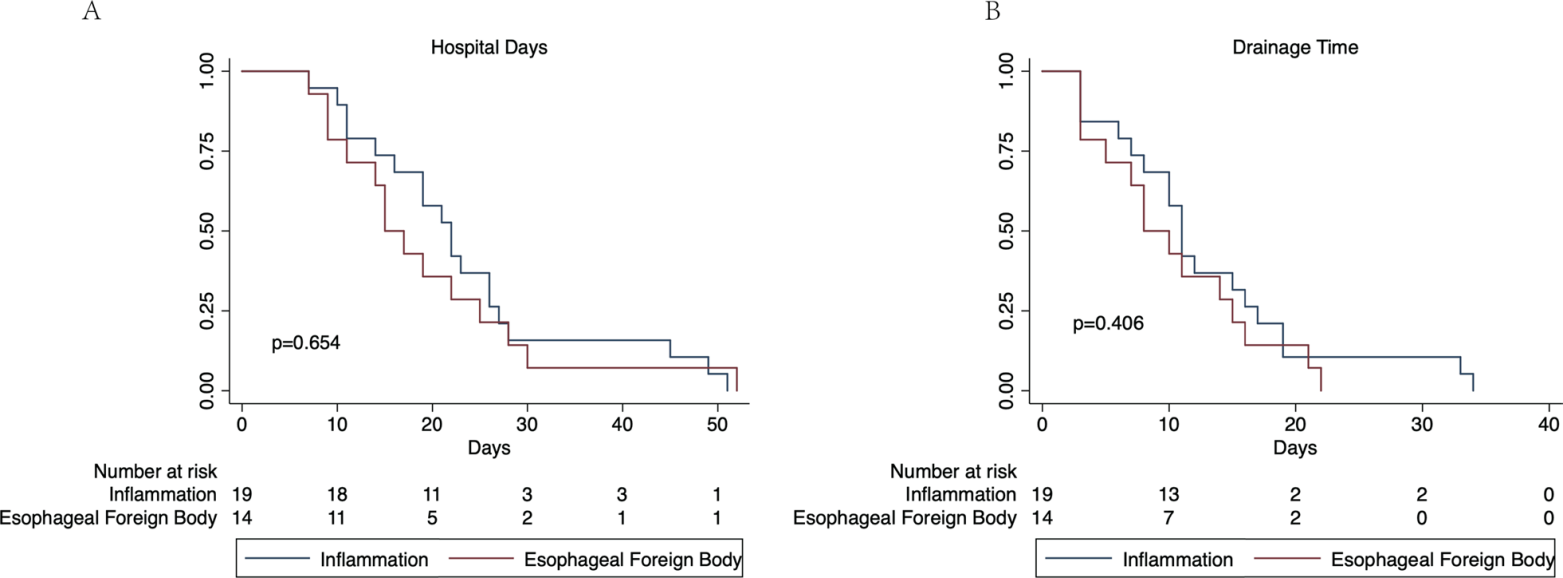
Survival analyses related to the number of hospital days and drainage time. **A** There was no significant difference in terms of hospital days between the two groups. **B** There was no significant difference between the two groups in terms of drainage time

We set up two survival analyses on account of hospital days and drainage time to evaluate differences between the two groups; there were no significant differences detected between the two groups (Fig. 2).

## Discussion

Neck abscess is a type of disease with a relatively low incidence, partly because most deep neck infections can be cured by conservative treatment. However, once formed, a neck abscess can result in serious consequences, including, but not limited to, airway obstruction, sepsis, mediastinitis, pericarditis, brain abscess, empyema, pneumonia, carotid artery erosion, and jugular vein thrombosis [2]. EFB is one of the most common diseases in E.N.T. emergencies. More than 24,000 cases of upper gastrointestinal foreign body impaction were reported within 5 years, and this data is likely to be underestimated [7]. Thus, the prognosis of such patients cannot be ignored. The probability of developing a neck abscess in EFB patients was observed carefully during our clinical experience. We considered EFB as an important inducement of neck abscesses, except for traditional inflammatory causes. To our knowledge, no previous study has considered the differences between patients with neck abscesses caused by esophageal foreign body impaction and by inflammatory disease.

In this study, we included 33 adult individuals who had undergone open surgeries for neck abscesses; these were divided into an EFB group (14 cases) and an inflammatory group (19 cases); no children were involved due to the lack of a pediatric department. Patients in the EFB group all had a definite history of foreign body intake and underwent endoscopic removal (failure occurred in three cases). The histories of the original inflammatory diseases were also identified in all patients in the inflammatory group except for one (unknown) in which a history of foreign body intake was excluded. Throat infection and odontogenic infection were the main causes of neck abscesses in the inflammatory group; this corresponded with previous literature. The diagnosis of each patient was confirmed by radiologists with more than ten years of experience from contrast-enhanced CT scans, according to current clinical protocols [8, 9]. In such cases, CT images showed extensive soft tissue swelling in different areas of the neck (in some cases, the mediastinum as well), with fluid and sometimes air foci in between. However, esophageal perforation cannot be ascertained through CT scans with merely a presentation of thickened esophagus and overflowing air. Thus, the CT images for the two groups were not specifically distinguishable unless a foreign body had been left. Esophageal perforations were confirmed through preoperative endoscopy (performed in all EFB cases) and intraoperative exploration. Recently, some researchers suggested that magnetic resonance imaging (MRI) can provide more detailed information relating to neck abscesses than contrast-enhanced CT, including retropharyngeal (RPE) and mediastinal edema (ME) [10, 11]. However, on account of the longer scanning time, higher expense, and lower applicability, an MRI test for every case was difficult to achieve. For example, it was certainly not realistic for a patient at a high risk of airway obstruction to tolerate this latency. The advantage of MRI for neck abscesses has yet to be fully proven. Therefore, we considered contrast-enhanced CT as the only imaging evidence.

Our results demonstrated that most patients endured a relatively long period from the onset of symptoms to surgery. This may be because our hospital is a comprehensive Grade 3A hospital with a state-of-the-art endoscopy center. Therefore, most patients with neck abscesses were transferred to our hospital from other medical institutions. Before the patients arrived at our hospital, some of those with inflammation had already received conservative treatment in other hospitals, which had failed; while the EFB patients had undergone endoscopic removal and remained home thereafter. Second, patients with deep neck infections first arrived at our hospital with classical symptoms such as fever, pharyngalgia and dysphagia; some presented with dyspnea. Except for those who required emergency operations, a series of clinical experiments and imaging examinations were required to clarify the diagnosis for most of the patients. Surgical treatment was arranged only when the presence of an abscess had been confirmed. In addition, five patients received ultrasound-guided puncture and drainage beforehand, which has become a popular means of treating small abscesses over recent years. Open surgery was used as a compensatory arrangement for these cases.

In general, patients in the two groups received the same form of therapy. They all underwent an incision to gain access to the neck abscess with the addition of mediastinotomy if necessary; post-surgery, the patients received anti-infection treatments. Postoperative negative pressure drainage was used in most cases (84.9%), and postoperative irrigation was also included in the treatment of 6 cases to promote the healing process. In case of postoperative dysphagia, all patients received nasal feeding, which was withdrawn prior to discharge. There were no significant differences between the groups with regard to the hospital stay, the drainage-tube removing time, the risk of ICU admission, and the probability of intubation and tracheotomy. Esophageal perforation was detected for each patient in the EFB group, suggesting that the infection infiltrated from esophagus to the neck soft tissue from the perforation. Notedly, we identified two cases of esophageal perforation in the inflammatory group, indicating that primary inflammatory diseases could invade the esophagus. However, except for esophageal perforation, the incidence of other preoperative or postoperative comorbidities did not differ significantly when compared between the two groups. It proved impracticable to repair the esophagus during abscess incision for patients with esophageal perforation due to the existence of acute inflammation; thus, each of them received nasal feeding following surgery. Routinely, a CT review was arranged for each patient to assess their postoperative recovery status after approximately two weeks. In addition, an iodine water esophagogram was arranged for cases involving esophageal perforation. A second-stage repair of the esophagus may be required if perforation is still evident in the esophagogram. However, the perforations usually all healed on their own after two weeks, and the patients developed the capability to eat normally without nasogastric tubes. Our findings indicate that esophageal perforation was not related to a progression in patients with neck abscesses; those with esophageal perforation only required incision and drainage with no need for second-stage repair of the esophagus.

According to multivariate analysis, longer duration from symptom onset to surgery increased the time taken to remove the drainage tube and the length of ICU stay, thus indicating that infection worsened with delayed treatment, as reported previously [12]. The adoption of mediastinotomy led to a longer hospital stay, implying that it became more time and money consuming when the infection affected the mediastinum. The mediastinal extension of infection was previously shown to be associated with a worse prognosis and a higher mortality rate [13]. Our current survival analyses showed that there were no significant differences between the two groups in terms of hospital days and drainage time.

To the best of our knowledge, this is the only study to investigate differences between patients with neck abscesses induced by EFB and inflammation. However, there are some limitations that cannot be ignored. First of all, a certain degree of bias was unavoidable due to the restriction of sample size caused by the low incidence of the disease and the fact that this study was based in a single center. For instance, the inflammatory group represented a higher proportion in many indicators (although this was not statistically significant), including hospital stay, the risk of ICU admission, and postoperative comorbidities. It is possible that the real actual relationships could not be detected due to the small sample size. In addition, due to the retrospective nature of this study, there is also the risk of bias with regard to inaccuracies in the clinical records. Therefore, retrospective, multi-center, and prospective studies with larger sample sizes are required in the future to fully identify the differences between neck abscesses induced by EFB and inflammation.

## Limitations

1. Due to the retrospective nature of this study, some bias was attributed to the inaccuracy of clinical records.
2. The sample size of the study was small because this was a single center retrospective study.

## Conclusion

To summarize, we discovered that patients with neck abscesses related to EFB or inflammation received the same form of therapy with no significant differences in terms of prognosis. Furthermore, esophageal perforation was irrelevant to the aggravation of neck abscesses, and there was no need for additional surgery to repair a perforated esophagus in patients with neck abscesses.

## Data Availability

The datasets could be found in the address as follow: Shen, Na (2022): Data for neck abscess. figshare. Dataset. https://doi.org/10.6084/m9.figshare.20200097.v1.
